# Sleep Characteristics of the Staff Working in a Pediatric Intensive Care Unit Based on a Survey

**DOI:** 10.3389/fped.2017.00288

**Published:** 2017-12-22

**Authors:** Yolanda Puerta, Mirian García, Elena Heras, Jesús López-Herce, Sarah N. Fernández, Santiago Mencía, Sleep Study Group, Alba M. Corchado

**Affiliations:** ^1^Pediatric Intensive Care Unit, General Gregorio Marañón Hospital, Madrid, Spain; ^2^Gregorio Marañón Hospital Research Institute, Madrid, Spain; ^3^Complutense University of Madrid, School of Medicine, Madrid, Spain

**Keywords:** sleep quality, sleep disorders, pediatric intensive care unit, health professionals, shift work

## Abstract

The objective is to evaluate the sleep characteristics of the staff working in a pediatric intensive care unit (PICU). They were asked to complete an anonymous survey concerning the characteristics and quality of their sleep, as well as the impact of sleep disturbances on their work and social life, assessed by Functional Outcomes of Sleep Questionnaire (FOSQ)-10 questionnaire. The response rate was 84.6% (85% females): 17% were doctors, 57% nurses, 23% nursing assistants, and 3% porters. 83.8% of them worked on fix shifts and 16.2% did 24-h shifts. 39.8% of workers considered that they had a good sleep quality and 39.8% considered it to be poor or bad. The score was good in 18.2% of the staff and bad in 81.8%. Night shift workers showed significantly worse sleep quality on both the objective and subjective evaluation. There was a weak concordance (kappa 0.267; *p* = 0.004) between the perceived quality of sleep and the FOSQ-10 evaluation. Sleep disorders affected their emotional state (30.2% of workers) and relationships or social life (22.6%). In conclusion, this study finds that a high percentage of health professionals from PICU suffer from sleep disorders that affect their personal and social life. This negative impact is significantly higher in night shift workers. Many health workers are not aware of their bad sleep quality.

## Introduction

Sleep is a basic human necessity and has many important biological functions. It is regulated by the sleep–wake cycle (1, 2). Multiple factors can affect sleep quality and the sleep–wake cycle: age, psychological problems, diseases, hormonal, environmental, sociocultural, or economic changes (3, 4).

People affected by an impaired sleep–wake cycle can experience physical and psychological disorders. The most prevalent are concentration problems, tiredness, daytime sleepiness, irritability and headache. Social and family life can also be affected by sleep disorders (5, 6).

Workers whose job includes working in different shifts, especially night shifts, often experience sleep difficulties such as fragmented sleep, difficulty in falling and staying asleep, and poor sleep quality (7–13). A disorder called shift work sleep-wake disorder has been identified in workers with severe sleepiness and insomnia related to their work schedule (9).

Health workers undergo two adverse situations: shift–work schedule and high assistance activity stress. Therefore, they are on greater risk of developing sleep disorders (14–17).

Some studies have analyzed sleep characteristics of the staff working in adult intensive care units (18), but none of them have analyzed these sleep problems in the professionals of a pediatric intensive care unit (PICU).

The objectives of this study are as follows: first of all, to evaluate sleep characteristics and quality of the staff working in a PICU and its social implications. Second, to compare sleep quality in different shift work status and professional categories. Finally, to analyze the concordance between the subjective appreciations of sleep quality and the results of the Functional Outcomes of Sleep Questionnaire (FOSQ)-10 questionnaire.

## Materials and Methods

A cross-sectional study was performed in March 2016, based on an anonymous and voluntary survey that was filled in by the staff of the PICU (Appendix S1 in Supplementary Material). The study was approved by the Bioethics Committee of General Gregorio Marañón Hospital.

The survey consisted of 21 questions, including demographic data and general information related to their job and work shifts. Questions were based on other sleep questionnaires as the Epworth Sleepiness Scale (19) and Pittsburgh Sleep Quality Index (20) (questions 4–8, 9). Questions 1–3 were added by researchers in order to assess the emotional sphere and the presence of job-related anxiety. Questions 12–21 correspond to the FOSQ-10 questionnaire (21–23). It is considered to be a subjective evaluation of sleep quality and it evaluates the excessive daytime sleepiness and its impact on daily life activities. The original FOSQ questionnaire consists of 30 questions divided into 5 subscales: general productivity (8 items), activity level (9 items), vigilance (7 items), social outcomes (2 items) and intimacy and sexual relationships (4 items). Each dimension can have a minimum value of 0 (utmost functional impact) and a maximum value of 24 (no impact). A final score lower than 18 corresponds to bad sleep quality. In our research, we have used the short version called FOSQ-10 (21–23), where each question can be punctuated from 0 to 4 depending on the grade of difficulty in achieving the items. The scoring scheme for the original version of the FOSQ was applied to the shorter instrument. To obtain the total score, a mean-weighted item score was first computed for those subscales with more than one item. This approach prevented the distortion of the score resulting from missing responses. The total score was derived by calculating the mean of the subscale scores and multiplying that mean by 5. The original language of the questionnaire is English but, because not every worker in our PICU speaks English, the questionnaire was translated into Spanish by a native English speaker who works in our PICU. We also followed the indications of previous publications that used a Spanish version of FOSQ (23).

After informing all PICU professionals about the start day of the study, some paper surveys were left at the nurse station, in a visible place on the desk. A postbox was set for a 1-month period to collect all the completed surveys.

Data were analyzed with IBM SPSS Statistics 22 package. In order to compare qualitative variables, Chi Square test and Fisher’s exact test were used (when percentage of expected frequency minor than 5 exceeded 20%), with a confidence interval of 95%. The Kolmogorov–Smirnov test was applied to test for a normal distribution of quantitative variables. Student’s *t*-test and Mann–Whitney’s *U* test were used to compare these variables according to their Kolmogorov–Smirnov distribution result. Correlation between quantitative variables was calculated with non-parametric Spearman Rho test. To evaluate the statistical concordance between subjective self-reported sleep quality and the results from the FOSQ-10 questionnaire, we used the Kappa index as coefficient of intra-class correlation. A *p*-value of <0.05 was considered to be statistically significant.

## Results

### General Characteristics of PICU Professionals

The survey was completed by 88 (84.6%) out of 104 PICU professionals. Mean age was 38.8 years (SD 11); median age was 36 years (IQR 30–48). 85% were women. The distribution as per professional categories was as follows: 15 (17%) doctors, 50 (57%) nurses, 20 (23%) nurse assistants, and 3 (3%) porters. 29% of the staff worked in the morning shift, 21.5% in the evening, 33.3% at night, 10.8% worked on rotating shifts and 24-h shifts, and 5.4% worked only 24-h shifts. Almost one-third (31.2%) of the employees were single, 40.9% married, 22.6% lived with a partner, and 3.2% were divorced. 79.5% of the workers had children.

15.5% of the staff said they had little or no professional satisfaction. 62% confessed they relived job experiences outside work often or very often.

### Sleep Characteristics

Most workers (74.2%) slept an average of 6–8 h, 13% of them slept 4–6 h, and 9.8% between 8 and 10 h. Sleep quality was considered good by 39.8% of staff members. The rest of workers (60.2%) considered they had poor sleep quality.

According to the results of the FOSQ-10 questionnaire, however, 18.2% of workers showed good sleep quality. The remaining 81.8% had poor sleep quality.

Regarding awakenings during sleep hours, 28% of workers had none, 9.7% had one, 30.1% had two, and 16.1% had three or more awakenings per night. About half (50.8%) of them denied having problems to fall asleep again.

About 12.9% of the staff took some sleeping medication (11% morning shift, 10% evening shift, and 20% night shift) (*p* = 0.733).

### Relation between Sleep Quality and Personal and Family Features

Table 1 shows the relationship between quality of sleep, age, and number of children.

**Table 1:** Relationship between sleep quality and personal and family characteristics.

	Functional Outcomes of Sleep Questionnaire-10			Subjective perception		
	Good quality	Poor quality	*p*	Good quality	Poor quality	*p*
Mean age (SD)	35.7 ± 11.6	39.6 ± 11	0.205	35.8 ± 9.1	40.8 ± 11.7	0.027
Number of children	0.53 ± 0.94	0.90 ± 0.93	0.139	0.54 ± 0.9	1 ± 0.92	0.02

*Workers who reported a bad perception of their sleep quality were older and had more children*.

There were no statistical differences in the FOSQ-10 evaluation regarding age or number of children. However, workers with a bad subjective perception of their sleep quality were older and had more children.

The perception of sleep quality and the results of the FOSQ-10 did not show any differences between men and women (*p* = 0.302).

### Sleep Quality Comparison between Professional Categories

**Figure 1:**
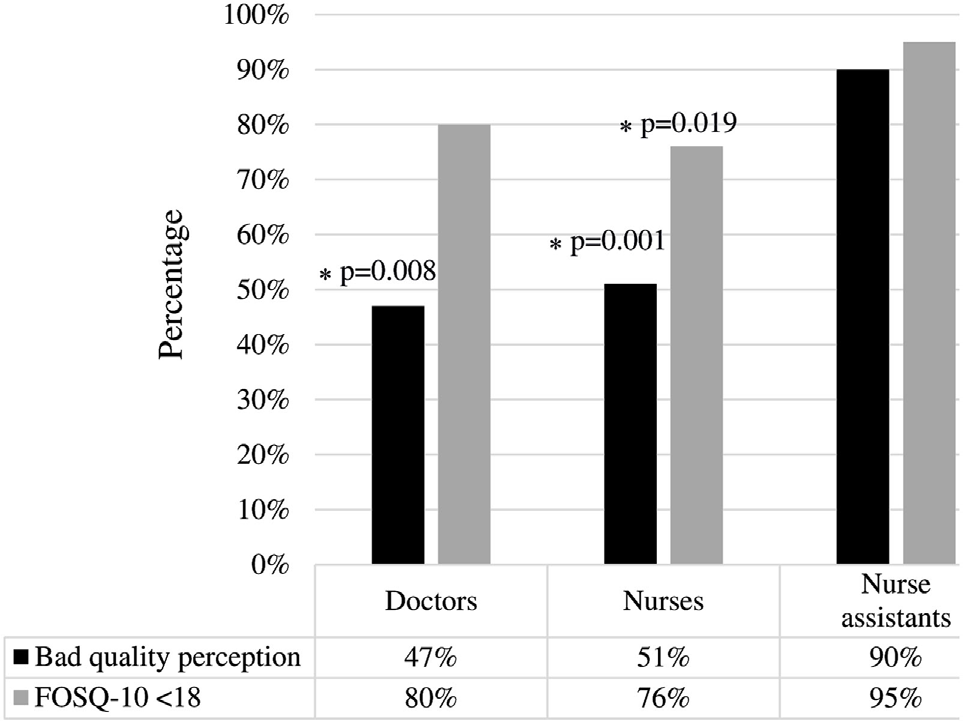
Sleep quality comparison between professional categories. Nurse assistants had worse sleep quality than the rest of workers, according to both perception and to the Functional Outcomes of Sleep Questionnaire (FOSQ)-10. *There is a significant difference between doctors and nurse assistants according to “poor-quality perception” (*p* = 0.08), and also between nurses vs nurse assistants according to either “poor-quality perception” or FOSQ-10 < 18 (*p* = 0.001 and *p* = 0.019).

Figure 1 shows the perception of sleep quality and the results of the FOSQ-10 questionnaire in the different professional categories.

Nurse assistants had worse sleep quality (both perception and FOSQ-10 scores) than doctors and nurses, although the difference between doctors and nurse assistants was only significant from the perception point of view. There were no significant differences between doctors and nurses in neither perception nor the FOSQ-10 questionnaire.

### Comparison of Sleep Quality between Work Shifts in Nurses

**Figure 2:**
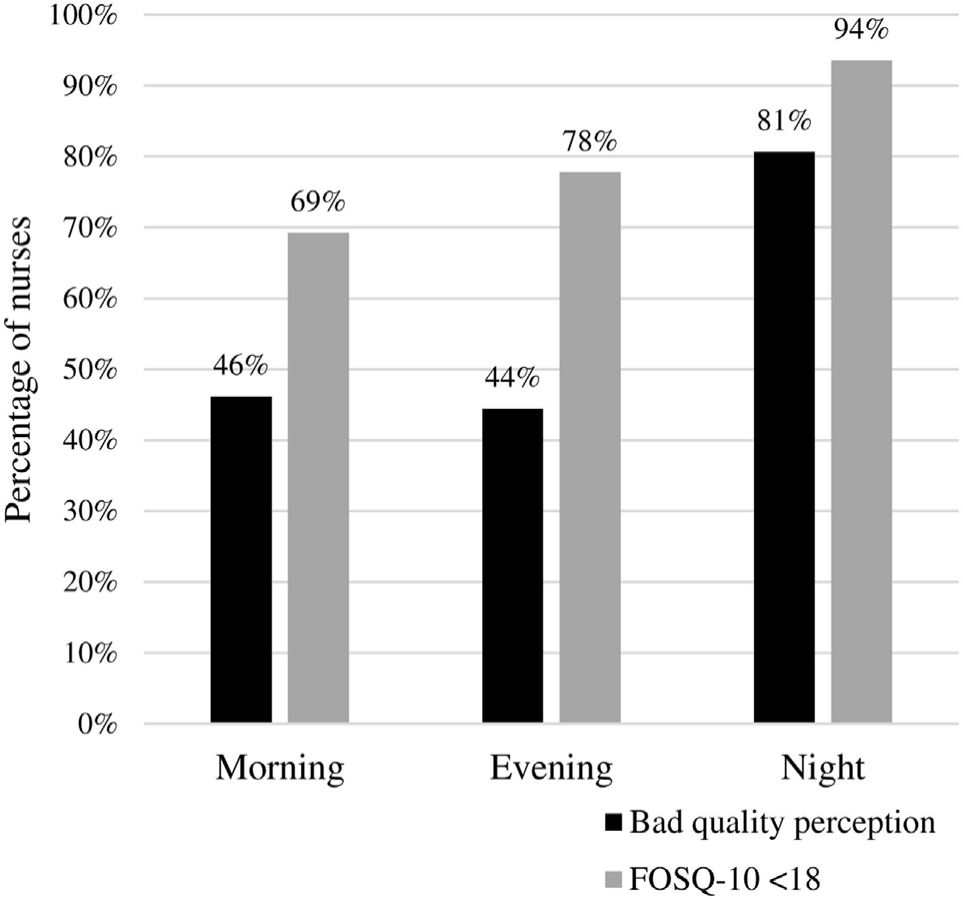
Sleep quality comparison between nurses’ work shifts. Night shift nurses had worse sleep quality (perception, *p* = 0.02; Functional Outcomes of Sleep Questionnaire-10, *p* = 0.05).

Figure 2 shows the comparison of sleep quality between nurses with different work shifts (morning, evening or night).

Night shift nurses had worse sleep quality (80.6% had bad perceived sleep quality and 93.5% had FOSQ-10 score <18) than nurses working mornings (46.1 and 69.2%, respectively) and evenings (44.4 and 77.7%, respectively). The comparison was statistically significant for both the perceived quality of sleep (*p* = 0.02) and the results of the FOSQ-10 (*p* = 0.05).

### Analysis of Possible Risk Factors for Bad Sleep Quality According to the FOSQ-10

Considering the results of the FOSQ-10 questionnaire, we identified some possible independent risk factors for bad quality of sleep: age (cut-off point of 40 years), having children, having a stable partner, labor seniority (more or less than 5 years working in the PICU), professional category (nurse assistants vs doctors and nurses), and work shift (morning and evening shifts vs nights). Results from the univariate analysis are shown in Table 2.

**Table 2:** Risk factors for bad sleep quality according to Functional Outcomes of Sleep Questionnaire (FOSQ)-10.

Risk factors		FOSQ-10 <18 (% workers)	*p*
Age	<40 years old	78.8	0.41
	>40 years old	86.5	
Having children	No	73.3	0.10
	Yes	88.9	
Having stable partner	No	69.7	0.05
	Yes	87.9	
Labor seniority	<5 years	78.0	0.59
	>5 years	83.7	
Professional category	Nurse assistants	95	0.10
	Doctors + nurses	76.9	
Work shift	Morning + evening	73.3	0.04
	Night	93.5	

*Working nights is the only significant risk factor*.

Statistically significant differences were only found for the “night shift” factor.

### Impacts of Sleep Disorders on Workers’ Daily Life

**Figure 3:**
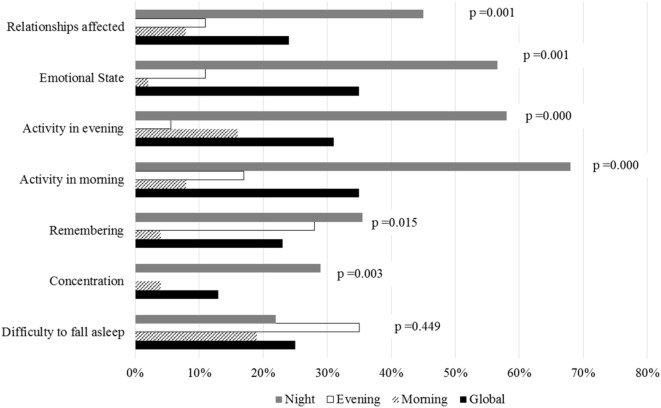
Comparison between work shifts of the impact of sleep disorders on daily life activities and relationships. Workers who had worse sleep quality according to Functional Outcomes of Sleep Questionnaire-10 also had a greater impact on their daily life activities.

Figure 3 shows the impact of sleep disorders on daily life activities and relationships according to the usual work shift.

Workers who had poor sleep quality scores in the FOSQ-10 questionnaire had a greater impact on their daily life. They had more concentration problems (*p* = 0.0001), trouble remembering (*p* = 0.0001), difficulty in visiting family and friends (*p* = 0.0001), trouble watching movies (*p* = 0.0001), difficulty in keeping active in the mornings and evenings (both *p* = 0.0001) and a greater impact on their emotional state (*p* = 0.0001). There were no significant differences regarding difficulty in falling asleep (*p* = 0.449) or difficulty in driving short or long distances (*p* = 0.164 and *p* = 0.278).

### Comparison and Concordance between the Perceived Sleep Quality and FOSQ-10 Questionnaire

The scores from the FOSQ-10 questionnaire showed a higher percentage of poor sleep quality than self-perception assessments (81.8 vs 60.2%, *p* < 0.001).

Concordance between FOSQ-10 scores and self-perception of sleep quality was low (kappa 0.267, *p* = 0.004) (Table 3). Regarding good sleep quality, concordance was 31.4% (35 workers referred to having the feeling of good sleep quality but only 10 of them showed so in the FOSQ-10 score). On the other hand, concordance for bad sleep quality was 90.5% (53 workers reported bad sleep quality and 48 of them had consistent scores in the FOSQ-10 questionnaire) (Table 3).

**Table 3:** Concordance between perceived sleep quality and Functional Outcomes of Sleep Questionnaire (FOSQ)-10 questionnaire for all workers.

	FOSQ-10		
Perceived sleep quality	Poor < 18	Good > 18	Total
Poor	48	5	53 (60.2%)
Good	24	11	35
Total	72 (81.8%)	16	88

*Concordance between the perceived sleep quality and FOSQ-10 questionnaire was low (kappa 0.267, *p* = 0.004)*.

*Concordance between both measures for good sleep quality was 31.4%, but it was higher (90.5%) for bad sleep quality*.

**Figure 4:**
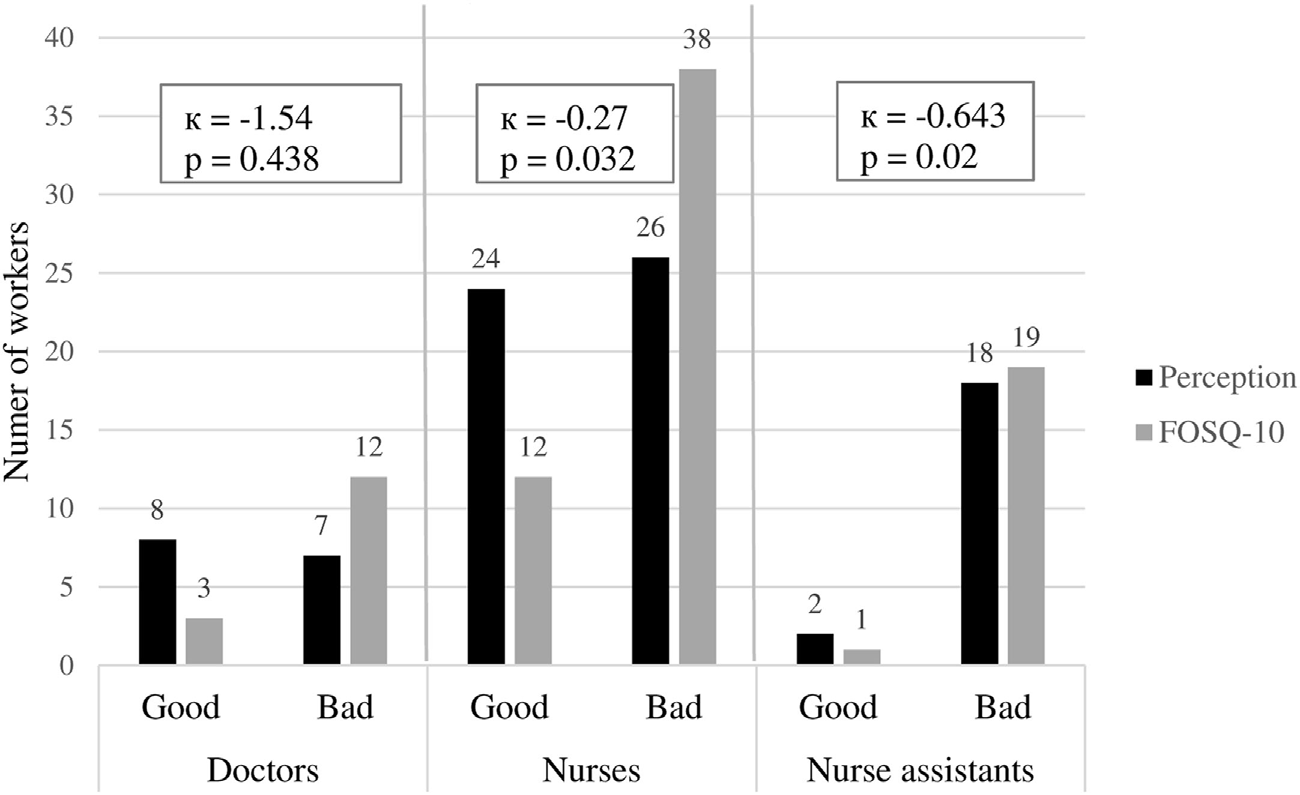
Concordance between perceived sleep quality and Functional Outcomes of Sleep Questionnaire (FOSQ)-10 questionnaire according to each professional category. The best concordance between perceived sleep quality and FOSQ-10 results was for the nurse assistants group.

Concordance between both evaluations of sleep quality in different professional categories is shown in Figure 4. Concordance was good in nurse assistants (Kappa = 0.643, *p* = 0.02), low in nurses (Kappa = 0.27, *p* = 0.032), and non-existent in doctors (*p* = 0.438).

## Discussion

This is the first study assessing sleep quality in a PICU, including all health professional categories. Participation was high, so it was possible to analyze this population quite reliably. Our results show that a very high percentage of PICU workers suffer from bad sleep quality. This percentage is higher than that in other professions scheduled by shifts (6, 9).

Some studies have analyzed sleep disorders in health professionals, mainly nurses, as well as its impact on daily life activities; however, none of them have studied sleep problems of the staff of PICUs (15–18).

In the case of PICUs, rotating shifts affect most workers (doctors, nurses, and nurse assistants). In this environment, multiple factors can trigger the onset of sleep problems. Sleep problems may worsen the burnout phenomenon described in critical care nurses (24, 25). Some of these stressful factors include physical and emotional stress of dealing with severely ill children, emotionally overwhelming situations concerning patients and their families, the need for permanent monitoring, permanent menace of the onset of life-threatening situations, procedures or techniques at any time, high light and noise intensity even at night, etc.

Sleep disorders have an important impact on daily life. Studies show that lack of sleep and poor-quality sleep have negative cognitive and behavioral effects. It impairs attention, alertness, concentration, reasoning, and problem solving, and leads to daytime sleepiness and poor overall performance (2, 5). When workers need to sleep during the daytime (due to their work shifts), their circadian rhythm may go out of synchrony. This affects sleep quality by decreasing slow sleep phases and increasing the REM phase (7).

Yong et al. (6) found that 36% of general night shift workers had problems on their daily life activities related to sleep disorders. However, when we studied the impact of sleep disorders on daily life and social relationships, we saw a significantly higher percentage of workers affected by sleep disorders. This may be due to the additional stress factors of working in a PICU that we mentioned before.

When comparing sleep quality between different work shifts, we see that working nights is one of the main risk factors for sleep disorders, problems on social and family life, and other health issues (5, 9).

In our study, night shift workers reported worse sleep quality and more repercussion on daily life activities. They had greater emotional instability and worse social relationships. These results are consistent with Ferri et al.’s (18) findings about nurses of different hospital units.

We also compared sleep quality between different professional categories, as we did not find any other previous studies comparing that. In our study, nurse assistants showed worse sleep quality than nurses and doctors. The reason for these differences remains unclear, but it may be due to personal life factors, family issues, or job schedules.

Other important point of our study was to analyze the concordance between perceived sleep quality and FOSQ-10 questionnaire. When comparing the workers appreciation of sleep quality with the FOSQ-10 scores, we found that the FOSQ-10 showed a significantly higher percentage of bad sleep quality (81.8 vs 60.2%; *p* < 0.05).

Concordance between perceived sleep quality and the FOSQ-10 questionnaire was very low (kappa = 0.267, *p* = 0.004). Concordance was very good when classifying bad sleep quality but poor when classifying good sleep quality. Workers who thought had bad sleep quality also had a bad FOSQ-10 score in most of the cases. However, more than half of the workers who thought had good sleep quality actually had a bad FOSQ-10 score.

We speculate that many workers are not aware of their bad sleep quality, probably because they are already used to their situation and consider it as normal.

In view of the above, it is necessary to develop awareness campaigns for health professionals working at intensive care units in order to raise consciousness about the importance of good sleep quality and its impact on professional performance and personal life.

New protocols, prevention programs, and strategies should be adopted to improve sleep quality and to optimize the resting time of workers (10, 26).

In the light of these findings, we would recommend creating new work schedules, avoiding night shifts longer than 8 h, including regular breaks within each work shift. It would not involve an additional economic expenditure, as the number of workers would remain the same. Improving environmental conditions is also important to promote circadian rhythm adaptation at both workstations (using bright and bluish lights) and out of work (optimizing room conditions and sleep hygiene at home to be able to sleep over the day; promoting relaxing activities; and improving the balance between family/social relationships, work, and sleep time, etc.).

We cannot say that our results are applicable to other units or workers in the hospital because the stress level and workload is different depending on the job post.

### Limitations

This is a single-center study, so our sample of people are under the same professional environment. Hence, more studies, including other intensive care units, are necessary to confirm our results.

Participation rate was very high in our study, but still the sample size in each of the professional groups was rather small, which can limit the statistical power of some analysis.

We must keep in mind that this is a descriptive study based on a survey and there may be many uncontrolled factors.

Finally, this study analyzes the influence that working in a PICU and shift works have over sleep quality. However, other important factors affecting sleep quality, such as emotional status, family situation or housing, have not been evaluated.

## Conclusion

We can say that a high percentage of health professionals working in a PICU suffer from bad sleep quality, which negatively impacts their social life and daily life activities. Many health professionals are not aware of their bad sleep quality. Night shift workers have worse sleep quality than those working on mornings or evenings.

## Ethics Statement

The study was approved by the Bioethics Committee of General Gregorio Marañón Hospital.

## Author Contributions

YP: data collection and manuscript drafting. MG: data collection and manuscript drafting. EH: study design data collection. JL-H: study design and progress supervision. SM: study design and progress supervision. SF: manuscript drafting and language revision.

## Conflict of Interest Statement

The authors declare that the research was conducted in the absence of any commercial or financial relationships that could be construed as a potential conflict of interest. The handling editor declared a past co-authorship with one of the authors JL-H.
