# Corrigendum: Sleep Characteristics of the Staff Working in a Pediatric Intensive Care Unit Based on a Survey

**DOI:** 10.3389/fped.2018.00287

**Published:** 2018-10-10

**Authors:** Yolanda Puerta, Mirian García, Elena Heras, Jesús López-Herce, Sarah N. Fernández, Santiago Mencía, Sleep Study Group, Alba M. Corchado

**Affiliations:** ^1^Pediatric Intensive Care Unit, General Gregorio Marañón Hospital, Madrid, Spain; ^2^Gregorio Marañón Hospital Research Institute, Madrid, Spain; ^3^Complutense University of Madrid, School of Medicine, Madrid, Spain

**Keywords:** sleep quality, sleep disorders, pediatric intensive care unit, health professionals, shift work

This study has been funded by Red SAMID. RETICS, ISCIII – Sub-DirectorateGeneral for Research Assessment and Promotion, and the European Regional Development Fund (ERDF), ref. RD 12/0026/0006.

The authors apologize for this error and state that this does not change the scientific conclusions of the article in any way.

## Conflict of interest statement

The authors declare that the research was conducted in the absence of any commercial or financial relationships that could be construed as a potential conflict of interest.

