# Supplementary material for: Sleep Characteristics of the Staff Working in a Pediatric Intensive Care Unit Based on a Survey
Source: Front Pediatr. 2017 Dec 22;5:288. doi: 10.3389/fped.2017.00288 (PMC5748084; doi:10.3389/fped.2017.00288)
Supplement: Supplementary file 1 [file Data_Sheet_1.docx]

Appendix 1

SLEEP QUALITY SURVEY FOR UCIP HEALTH PROFESSIONALS

Date:

Gender:

Age:

Civil Status:

- Single
- Married
- Divorced
- Widow
- Living as a couple
- Others (specify)…………..

Number of children:

- Age of children:

Professional category in PICU:

Number of working hours per week:

Work shift in PICU:

- Morning
- Evening
- Night
- On-call shifts (17 h and 24 h)

For how long have you been working in a PICU?

1. Do you feel satisfied with your job?

- Not at all
- Yes, a little
- Yes, quite a lot
- Yes, very much

1. Do you practice any activity related to your job out of hours?
   - Yes
   - No
2. Do you usually remember any situations experienced at work out of the hospital?
   - No, never
   - Yes, sometimes
   - Yes, quite often
   - Yes, very often
3. How many hours do you usually sleep per day?
4. How would you rate your sleep quality overall?
   - Very good
   - Good
   - Normal
   - Bad
   - Very bad
5. Do you have difficulty falling asleep?
   - No, never
   - Yes, a little
   - Yes, quite a lot
   - Yes, very much
6. Do you usually awake during the night?
   - Yes (How many times?....................)
   - No
7. In case you said yes, is it difficult for you to fall asleep again?
   - Yes
   - No
8. Do you usually receive any treatment?
   - Yes (Which one?..........................................................)
   - No
9. Do you usually take any medication to sleep?
   - Yes (Which one?...........................................)
   - No
10. Do you usually take stimulating drinks or substances (coffee, tea, Coke., etc)?
    - Yes (Which one? How often?..................................)
    - No

**FOSQ-10**

1. Do you have difficulty concentrating on the things you do because you are sleepy or tired?
   - Yes, extreme
   - Yes, moderate
   - Yes, a little
   - No
2. Do you generally have difficulty remembering things because you are sleepy or tired?
   - Yes, extreme
   - Yes, moderate
   - Yes, a little
   - No
3. Do you have difficulty operating a motor vehicle for short distances (less than 50Km) because you become sleepy?
   - Yes, extreme
   - Yes, moderate
   - Yes, a little
   - No
4. Do you have difficulty operating a motor vehicle for long distances (greater than 50 Km) because you become sleepy?
   - Yes, extreme
   - Yes, moderate
   - Yes, a little
   - No
5. Do you have difficulty visiting your family your family or friends in their home because you become sleepy or tired?
   - Yes, extreme
   - Yes, moderate
   - Yes, a little
   - No
6. Has your relationship with family, friends or work colleagues been affected because you are sleepy or tired?
   - Yes, extreme
   - Yes, moderate
   - Yes, a little
   - No
7. Do you have difficulty watching a movie or video because you become sleepy or tired?
   - Yes, extreme
   - Yes, moderate
   - Yes, a little
   - No
8. Do you have difficulty being as active as you want to be in the evening because you are sleepy or tired?
   - Yes, extreme
   - Yes, moderate
   - Yes, a little
   - No
9. Do you have difficulty being as active as you want to be in the morning because you are sleepy or tired?
   - Yes, extreme
   - Yes, moderate
   - Yes, a little
   - No
10. Has your mood been affected because you are sleepy or tired?
    - Yes, extreme
    - Yes, moderate
    - Yes, a little
    - No
